# An overview of radioactive waste disposal procedures of a nuclear medicine department

**DOI:** 10.4103/0971-6203.79692

**Published:** 2011

**Authors:** R. Ravichandran, J. P. Binukumar, Rajan Sreeram, L. S. Arunkumar

**Affiliations:** Medical Physics Unit, National Oncology Center, Muscat, Sultanate of Oman; 1 Royal Hospital, Muscat, Sultanate of Oman; 2 DGEA, Ministry of Health, Muscat, Sultanate of Oman

**Keywords:** Delay tanks, iodine-131, isolation wards, radioactive wastes

## Abstract

Radioactive wastes from hospitals form one of the various types of urban wastes, which are managed in developed countries in a safe and organized way. In countries where growth of nuclear medicine services are envisaged, implementations of existing regulatory policies and guidelines in hospitals in terms of handling of radioactive materials used in the treatment of patients need a good model. To address this issue, a brief description of the methods is presented. A designed prototype waste storage trolley is found to be of great help in decaying the I-131 solid wastes from wards before releasing to waste treatment plant of the city. Two delay tanks with collection time of about 2 months and delay time of 2 months alternately result in 6 releases of urine toilet effluents to the sewage treatment plant (STP) of the hospital annually. Samples of effluents collected at releasing time documented radioactive releases of I-131 much below recommended levels of bi-monthly release. External counting of samples showed good statistical correlation with calculated values. An overview of safe procedures for radioactive waste disposal is presented.

## Introduction

Medical applications of radioactive isotopes form one of the important peaceful uses of atomic energy. Unsealed radioactive isotopes are used in hospitals for diagnostic and therapeutic applications in various health disorders. Safe use of radioisotopes in medical applications is the main issue in obtaining clearance from national regulatory authorities. The important issues are 1) safe custody of the received radioisotopes, 2) surveillance for their safe applications in the department and 3) the disposal of the radioactive wastes generated from human use of these radioisotopes. The issues relating to management of radioactive wastes, are very well formulated internationally, and guidelines for radioactive waste disposal are well documented.[1–8] The radioactive waste disposals must take into account permissible concentrations applicable from the standpoint of community safety, ensure that the degree of dilution envisaged is achieved at the discharge point (from the institution into the sewage system), and the hazard to the general population is insignificant in the event of the sludge containing radioactive waste material being used as fertilizer. Radioactive waste from nuclear medicine procedures can be dealt with either by simply storing the wastes safely till radioactive decay reduces the activity to a safe level or possibly by disposal of low-activity waste into the sewage system. A controlled disposal is defined as disposal with permission from the regulatory authority and appropriate monitoring. Oman being a newly formed member state under International Atomic Energy Agency (IAEA), there is a need for the local regulatory authority in Oman {Radiation Protection Adviser (RPA), Ministry of Health (MOH)} to review the records about the overall management of radioactive wastes. This paper outlines the various issues addressed by us in this regard.

## Materials and Methods

### Details of radioactivity handled

Radioactive sources received from GE Healthcare Buchler (Amersham, UK) or from Australia are used in this hospital only by the Department of Nuclear Medicine, which is located in the National Oncology Center complex of the hospital. Imaging procedures are carried out with Technicium-99m (^99m^Tc) formulations; and a limited number of procedures, using gallium-67 (^67^Ga) and m-Iodobenzyl guanidine (MIBG) (^131^I). The outlets of the toilets of the patients undergoing diagnostic procedures are connected to the delay tank meant for collection of radioactive iodine (^131^I) from the isolation rooms meant for these treatments. Two isolation rooms are located in the oncology ward of the Royal Hospital.

The radioactive consignments are received from GE Health Care Buchler, Amersham, UK; or from Australia,; after the clearances for import of radioactive isotopes are obtained from the Ministry of Environment (MOE) and RPA, MOH. ^131^I treatments have been started from February, 2006. Till the beginning of 2009, patients for the treatment of thyrotoxicosis received therapy as in-patients in the isolation rooms, in addition to the patients treated for postoperative thyroid carcinoma. These patients were discharged generally after 72 hours or after their exposure rates at distance of 1 metrefall below 10 μSv/h. Based on international recommendations, it is generally accepted that[4,9] an amount of 45% and 90% of administered activity could be assumed to be present in the collected urine from patients of thyrotoxicosis and carcinoma thyroid, respectively, during their isolation period.

### Waste management

#### Diagnostic wastes

Procedures for handling and disposal of wastes generated from diagnostic use of radioactive isotopes are described in the Royal Hospital document.[10] All ^99m^Tc daily wastes (disposables) are allowed to mix with normal wastes after a 48-hour delay. All wastes like used syringes and gloves are collected in plastic containers with dates of collection being recorded. Used syringes of ^99m^Tc, ^67^Ga, ^131^MIBG are generally put in different containers. Syringes for long - lived radioactive isotopes undergo minimum of 2-months decay before these are released after monitoring by the department’s medical physicist using an end window Geiger Muller (GM) survey meter. Records of wastes released are maintained by the department. Details regarding any waste released to the Medical Wastes Treatment Plant (MWTP), MOH, Al Amerat, are entered in a pro-forma sheet, to be faxed with a copy to the RPA, MOH. Toilet wastes from diagnostic patients go into the delay tank to *increase volume* of therapeutic I-131 wastes from the isolation wards, thereby achieving higher dilution factor.

#### I-131 therapy wastes

**Figure 1:**
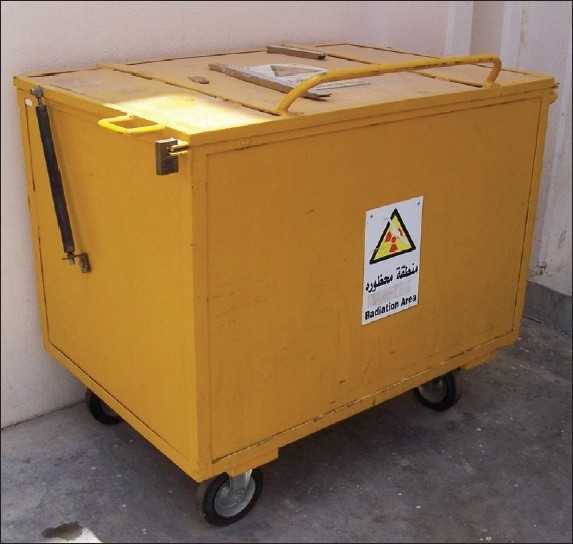
Temporary storage trolley in use for collection of solid wastes from I-131 wares in polythene bags. This trolley measurinig 1.20 × 0.85 × 0.80 m with castor wheels and locking facility, is anchored to the floor and is available at the waste collection yard, located behind the hospital premises

Iodine-131 in the form of capsule is the only radioisotope presently used for therapy in Royal Hospital. Unused capsules are maintained safely in stores and an inventory is maintained. The generated solid wastes from the isolation wards (yellow and black bags) are labeled with patients’ numbers and sent to a temporary storage trolley (TST), designed and fabricated locally for this purpose. TST is a lockable type [Figure 1] trolley and is kept well protected in the backyard of the hospital, and the contents are allowed to decay for 2 to 3 months before disposal. Individual bags are monitored by contamination monitor, certified and then disposed. The pro-forma sheet, described earlier is filled and faxed for the MWTP records.

#### Delay tanks

**Figure 2:**
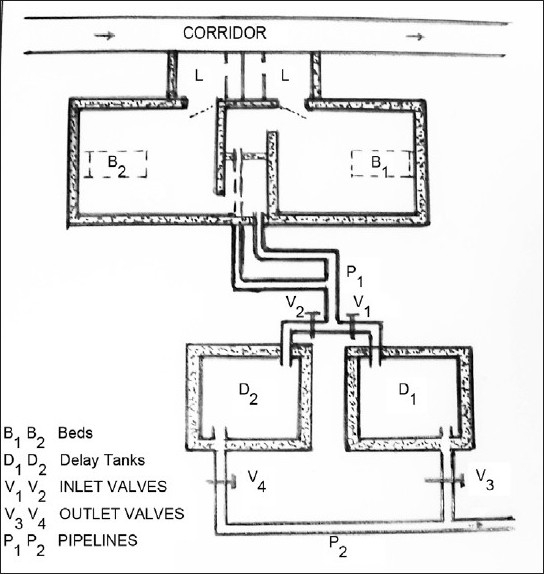
Schematic view of iodine-131 isolation rooms 1 and 2 (B1, B2) and delay tanks D1 and D2. V1, V2 are inlet valves and V3, V4 are outlet valves (Details about volumes of delay tank and monitoring system have been given in the text)

Liquid wastes (outlets from isolation room toilets) generated from ^131^I administrations are collected in the delay tank. The principle of delay tank system for I-131 effluents is explained in Figure 2. Two isolation rooms have sewage connections to twin concrete tanks located in the garden area below ground level. Both the tanks have a size of 5 × 4 × 2 m each with 40 kL individual volumes. Total capacity of filling is 75% of volume, viz. 30 kL. Two submersible pumps are installed at 50 cm from the bottom of tanks. Level monitoring is done by ultrasound system. Float switches for monitoring effluent levels are kept at a height of 60 and 180 cm, respectively making the flush volume of delay tank system to be 24 kL. Business management system (BMS) shows percentage volume of effluents, along with a hooter alarm. An electronic control system exists at the engineering department to monitor the effluent levels in the delay tanks, status of opening – and closing of valves. Before the collected effluents are emptied, certification procedure is followed for filling of delay tanks, closing, clearing of the tanks.[11,12] During filling phase of one tank, the other tank in filled condition decays for about 2 months. Documents for filling, closing, emptying, radioactivity monitoring in released effluents are maintained in the nuclear medicine and engineering departments. The operational guideline followed for maximum limit of discharge is 3.7 MBq /d or an average monthly concentration of 22.2 MBq /m^3^ for I-131.

#### Procedure for release of delay tank effluents

Fifty milliliters of effluent sample is taken out from the delay tank which is due for release and checked by an end-window contamination monitor. Activity one milliliter of this sample is measured (counted) by single-channel analyzer spectrometer (Atom Lab, USA). One milliliter of delay tank sample and 1 mL of tap water are counted for 300 seconds by a Sodium Iodide (NaI) well counter at I-131 window. Each time, the well detector is calibrated by Cs-137 check source. The counting efficiency of the system is 30%. The measured excess counts over tap water sample are expressed as activity of the sample {disintegration per sec (dps)}.

## Results

Table 1 shows the total amount of activity (GBq) handled in the past. It can be seen from Table 1 that activity of ^131^I in 2009 increased by more than 50% as compared to the total activity used during 2008. Table 2 shows the breakup of the details of administered activities of ^131^I. In thyrotoxicosis treatments, the administered activities ranged from 479 to 627 MBq, with a mean of 574.7 ± 27.3 MBq (*n*= 50). For Ca. thyroid treatments, the used activities were in the range of 2.04 to 9.3 GBq, with a mean of 4.376±1.15 GBq (*n*= 70). It can be seen that in more number of patients, higher amount of activities (> 5 GBq) were administered. During the period 2006 – 2009, a total amount of 306.3 GBq (8,280 mCi) was administered in our hospital.

**Table 1:** Details of utilization of activity in the department

*Radio-isotope*	*Amount of activity used in each year GBq*					
	*2005*	*2006*	*2007*	*2008*	*2009*
Mo-99	540	575	695	735	423.5
Ga-67	3.7	8.8	8.1	8.1	16.5
I-131MIBG	0.39	0.62	0.52	1.07	0.59
I-131capsules	–	33.1	65.3	87.8	138.3

**Table 2:** Details of administered activity for ^131^I treatments

*Activity used in treatments*	*Number of patients for years*				
	*2006*	*2007*	*2008*	*2009*
Therapy for Ca. thyroid				
< 5 GBq	7	9	21	12
> 5 GBq	–	4	4	13
Thyrotoxicosis				
< 550 MBq	1	3	1	2
> 550 MBq	11	20	11	1

Table 3 shows a representative collection phase at the delay tank no.2. The growths of I-131activity with time from the start of the tank filling, activity at the time of closing and activity remnant at the time of release of the tank are shown in this table. Table 4 shows a summary of the number of days of filling, delay achieved till next tank filling, and the activity disposed. The estimated activity in released radioactive waste effluent from the delay tank, by gamma ray spectrometer well counting, agreed well (within 5 MBq tolerance in 17 out of 20 instances) with the activity obtained by theoretical calculation. During the month of June 2007, due to ‘Gonu’ cyclone, there was a sudden onset of heavy rains and there was need to release activity in 35 days instead of the usual 60 - 70 days.

**Table 3:** Representation of activity growth in delay tank during one filling

*Patient Sl. no.*	*Administered activity to the patient (MBq)*	*Period from start of delay tank filling (days)*	*Residual activity at the time of next addition (MBq)*	*New activity reaching the tank (MBq)*	*Cumulative activity on date of addition of new activity(MBq)*
1	3830	0	0	3450	3450
2	3830	34	185	3450	3635
3	3830	41	1989	3450	5439
4	3830	48	2977	3450	6427
5	3830	55	3518	3450	6968
6	3830	62	3814	3450	7264
7	569	62	7264	290	7554
	Closing tank	70	—	—	3777
	Delay phase	140	—	Releasing	18

**Table 4:** Details of filling, delay phases and activity released from delay tank

*Dates of closing delay tank*	*Total number of day*		*Residual activity MBq at closing of tank*	*Activity released MBq*	
	*Filling*	*Decay*		*Calculated*	*Measured*
1-1-2006	123	55	20.3	0.2	0.0
3-5-2006	73	51	80.4	1.0	6.5
15-7-2006	63	78	3922.4	4.6	0.0
16-9-2006	78	51	314.7	3.8	0.0
3-12-2006	51	57	7223.2	79.8	84.5
24-1-2007	57	72	1773.8	3.5	2.8
24-3-2007	72	35	4037.6	194.7	145.8
4-6-2007	35	60	2653.9	14.7	1.5
10-7-2007	60	75	Gonu	2.8	12.1
10-9-2007	75	86	1845.8	1.6	6.0
25-11-2007	86	75	2762.1	5.2	0.0
19-2-2008	75	70	2660.3	10.6	4.0
4-5-2008	70	57	4543.5	7.5	7.4
14-7-2008	57	68	1046.6	5.1	7.7
8-9-2008	68	109	1834.0	0.4	0.0
16-11-2008	109	57	7161.0	54.0	55.9
1-3-2009	57	98	1530.0	0.5	0.0
8-6-2009	98	59	3850.0	8.5	0.0
7-10-2009	59	69	1418.0	7.2	4.8
23-1-2010	69	48	2826.0	37.6	118.0

## Discussion

We have reviewed the data on the waste disposal methods followed in Royal Hospital. All the details provided may be applied to any other similar hospital. The presence of decaying food material mixed in yellow bags and presence of radioactive tissues in black bags created difficulties in storing these bags in storage rooms in isolation wards. In a few occasions, there was infestation of fruit flies, leading to problems in the isolation wards, in turn resulting in problems related to new admissions of patients. The sanitary gauze containing blood, or ‘breast-feeding-stopped patients’ milk pads sometimes gave rise to high residual activity in waste bags, which needed more time to come to background level. As a remedy to this problem, we have come out with an innovative solution, viz. to have a TST as described earlier. The locally designed TST has provided a solution for storage of solid wastes, and insecticide spraying could be periodically undertaken.

From Table 4, it can be observed that the amount of excretions indicated by the theoretical model correlates well (within 5 MBq tolerance in 17 out of 20 instances) to the released ^131^I into sewage system. In 202 patients treated for Ca. thyroid with radioactive iodine, Driver and Packer[1] found that 55% of administered activity is excreted in first 24 hours and 85% of the administered activity is discharged in the sewer over a typical patient-stay period of 5 days. The need for maintaining proper pro-forma sheets by the Radiation Safety Officer (RSO), giving details of storage and disposal of radioactive waste, was highlighted by these authors. In view of our data, we would like to recommend that there is no need for laborious counting of effluent samples, and only the delay/decay patterns need to be followed, because all the details of administered activity and estimated activity released from patients are available.

The last column in Table 4 indicates the actual amount of ^131^I radioactive waste released from the institution during the period of 4 years (20 instances). For environmental departments and regulatory governmental authorities, this will provide an accurate account of radioactivity actually released into the sewage treatment plant. Such data of released waste activity against administered activity is not reported in any earlier studies found in literature. In a tank of 30 kL volume, the theoretically estimated activity being in agreement within 5 MBq (135 μCi) of counted activity indicates that theoretical approximation is reasonably accurate.

From Table 4, it can also be observed that, mean delay time could not be extended for more than 60 days, because the other tank reaches its full capacity, necessitating release from it. There was an occasion during ‘Gonu’ cyclone in Oman, when there was onset of heavy rains and back pressure in toilets in isolation wards. This necessitated release of effluents within 35 days, and the counted ^131^I in the effluents was at highest level: however it was less than 22 MBq/ m^3^ operational limits. Recently IAEA has given a position statement - that there is no need for storage of urine in delay tanks and continuous sewage dilutions are sufficient.[13] However, in most of the places in the Middle East Asian countries, there are no centralized sewage management systems connected to hospitals and therefore such delay tanks appear to have a role in the management of radioactive urine in such places.

The main objective of this report is to highlight that the kinetic model of activity released from patients seems to be reasonably accurate; therefore, it appears that we can as well do away with laborious counting of inspection samples each time. This paper also documents that the released radioactive wastes were much below permitted activity levels of 22 MBq/ m^3^ for ^131^I{ (maximum released ^131^I activity 145.8 MBq / 36 m^3^) (4.5 MBq /m^3^) in the last 4 years, which will reduce concern among the general public about released radioactive wastes from hospitals.
